# Quantitative wood anatomy of *Juniperus excelsa* from Lebanon as a potential hydroclimate archive

**DOI:** 10.3389/fpls.2025.1558570

**Published:** 2025-08-29

**Authors:** Mansour Mdawar, Daniel Balanzategui, Ramzi Touchan, Emanuele Ziaco, Isabel Dorado-Liñán, Gerhard Helle, Ingo Heinrich

**Affiliations:** ^1^ Section 4.6: Geomorphology, German Research Centre for Geosciences, Potsdam, Germany; ^2^ Geography Department, Humboldt University Berlin, Berlin, Germany; ^3^ Department of Natural Sciences, German Archaeological Institute, Berlin, Germany; ^4^ Laboratory of Tree-Ring Research, University of Arizona, Tucson, AZ, United States; ^5^ Department of Geography, Johannes Gutenberg University Mainz, Mainz, Germany; ^6^ Departamento de Sistemas y Recursos Naturales, Universidad Politécnica de Madrid, Madrid, Spain

**Keywords:** dendroclimatology, juniper, palaeoclimatology, Middle East, drought, tree rings

## Abstract

The Middle East and North Africa is a hotspot for negative climate change impacts and potentially for conflicts over water resources. To protect future generations from destabilization and marginalization, governments need to consider the impact of climate change on water management issues. Long-term hydroclimatic information is needed for a reliable management of the water resources. However, only a few continuous high-quality meteorological records exist in MENA starting in the early 1900s, while the majority of existing records cover just the second half of the twentieth century, hence alternative sources such as tree-ring proxies to describe past climate dynamics will be a valuable add-on. Tree-ring width records of Greek juniper (*Juniperus excelsa* M. Bieb) have already been demonstrated to be useful for extending existing instrumental climate records. For the first time, we investigated the dendroclimatological potential of *J. excelsa* growing in the Lebanese mountains, focusing, also for the first time, on quantitative wood anatomy. We measured cell lumen diameter in radial direction and cell wall thickness in tangential direction for the tree rings formed during the years 1963 to 2019. The measurements were then correlated with monthly and seasonal climate records. Strongest correlations were found between lumen diameter and current May precipitation (positive) as well as maximum temperature (negative). Lumen diameter also exhibited significant correlations with drought during May and July to September). Climate correlations with tree-ring width were generally less significant. The study showed, for the first time, declining trends in the cell lumen and cell wall data since the early 1990s suggesting that in the Lebanese Mountains this important tree species *J. excelsa* seems to have been under increasing drought stress. The preliminary findings highlight the feasibility of building long chronologies of quantitative wood anatomical parameters for *J. excelsa* from the Lebanese mountains and the potential of such measurements as a paleoclimate archive, especially when focusing on water availability and drought patterns.

## 1 Introduction

The Middle East and Africa north of the Sahel (MENA) region is generally recognized as a major hotspot for climate change impacts often resulting in societal challenges (Sowers et al., 2011; García-Ruiz et al., 2011; Kubin et al., 2023) as well as political conflicts (Lange, 2019; Ozturk et al., 2015; Pal and Eltahir, 2016; Tuel and Eltahir, 2020; Waha et al., 2017). Large parts of present-day MENA region experienced such detrimental climatic and environmental changes repeatedly in the past, e.g., Fleitmann et al. (2022). A more detailed comprehension of the regional past climate dynamics is important, since it has been hypothesized that environmental change together with loss or gain of societal resilience played a significant role in conditioning societal expansions, contractions, and possibly even extinctions in this region e.g (Büntgen et al., 2011; Degroot et al., 2021; Scheffer et al., 2021; Matloubkari and Shaikh Baikloo Islam, 2022). Reliable regional climatic reconstructions provide crucial background information when studying the transformations within human societies and entanglement mechanisms in MENA more comprehensively. Such reconstructions can help to identify unprecedented declining trends such as river flow of the Euphrates River which then become crucial information for improved resource management as has been suggested recently, i.e. Abdelmohsen et al. (2022) and Zittis et al. (2022).

In many parts of the world, tree-ring-based climate reconstructions have been accomplished successfully (e.g., PAGES2k Consortium et al., 2017; Wilson et al., 2007). Also in the MENA region tree rings have demonstrated to be useful high-resolution climate proxies (Gebrekirstos et al., 2009; Sass-Klaassen et al., 2008; Wils et al., 2011; Gustafson & Speer, 2022; Sharifazari et al., 2023). Among several tree species, *Juniperus excelsa* has been shown to be one of the most suitable for dendroclimatology, likely because it is a long-living species and its wood remains are prominent in archaeological sites which facilitates constructions of long chronologies and climate reconstructions. In particular, juniper from the mountains in Turkey (Kuniholm et al., 2014), Jordan (Touchan et al., 1999), in Oman and Ethiopia (Sass-Klaassen et al., 2008; Wils et al., 2011) showed strong dendroclimatic potential. Further successful dendroclimatic studies were developed in Turkey, Bulgaria and Greece (Akkemik and Aras, 2005; Heinrich et al., 2013; Hughes et al., 2010; Köse et al., 2011; Touchan et al., 2007; 2005).

*J. excelsa* is one of the most widely used species for dendroclimatic research in the MENA region, where it is widely distributed and reaches multi-centennial ages, in particular in mountainous terrain of the Taurus and Lebanese mountains. Its ring-width chronologies are typically sensitive to climate. It is therefore an ideal target species for testing the potential of cellular level dendroclimatology (von Arx et al., 2016). The main goal of this explorative study was to assess the utility of wood anatomy of *J. excelsa* for dendroclimatic reconstructions with particular focus on MENA region climate (Balanzategui et al., 2021; Björklund et al., 2023; Lopez-Saez et al., 2023). Specifically we wanted to test the temporal and spatial climate relationships of intra-annual xylem anatomical parameters in *J. excelsa*. To achieve this goal, we developed ring-sector chronologies and assessed the strength of the climatic signal encoded in their wood anatomical structures.

## 2 Materials and methods

### 2.1 Location

**Figure 1:**
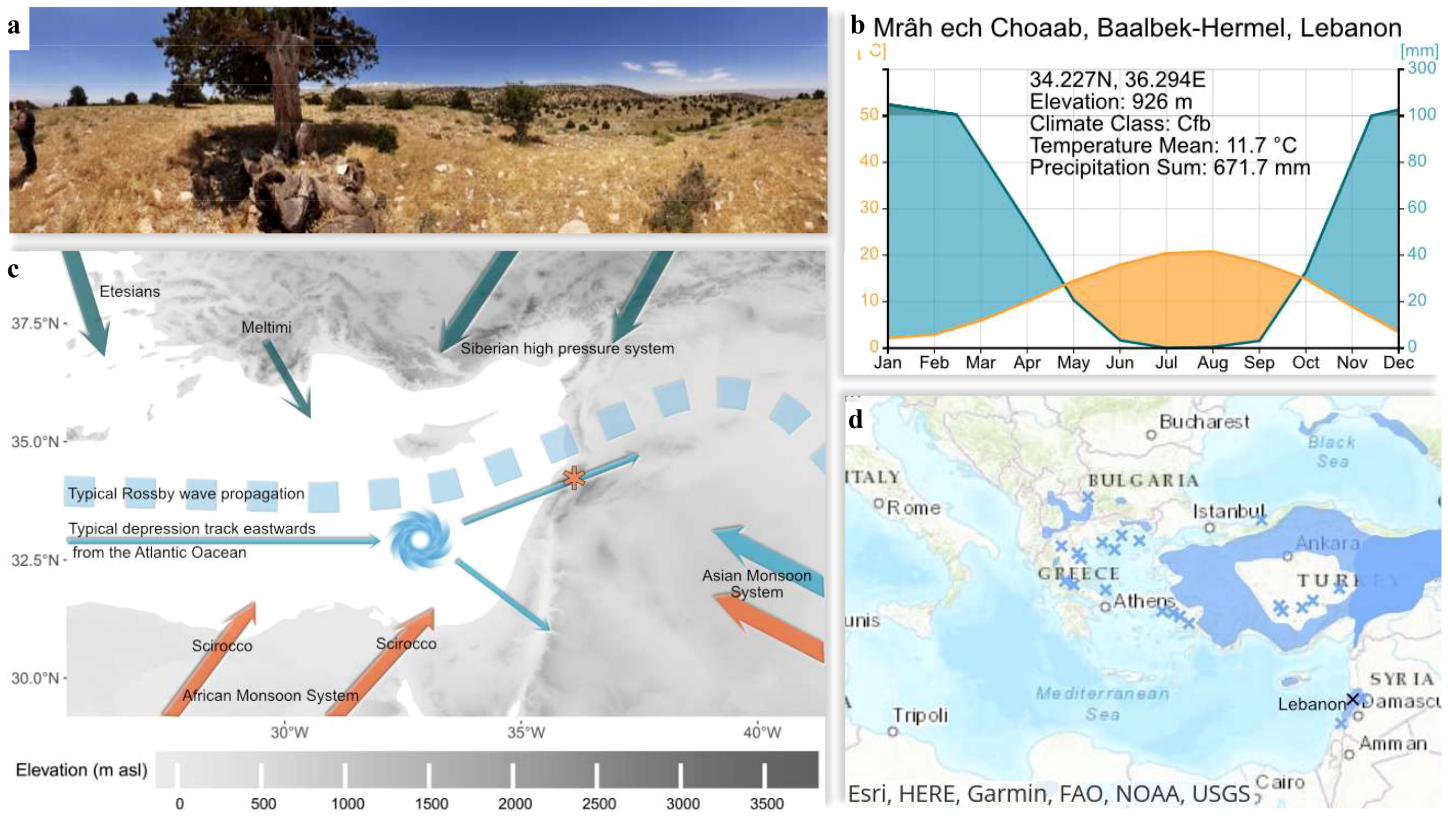
Geography of the study site, **(a)** a photograph of the site facing west, **(b)** Climate diagram (Years: 1950-2019, Data Source: CRU Time Series v4.04, ClimateCharts.net), **(c)** elevation map with main climate drivers of the area (the * is the site location), **(d)** geographic region with *Juniperus excelsa* distribution in dark blue shading or cross (the black x is the site position).

The study was conducted in Lebanon, on the leeward side of Mount al-Qurnat as-Sauda, the highest peak of Lebanon (3088m a.s.l., **Figure 1**), situated in the western mountain range of the country (34°12’14”N; 36°08’39”E). Located in the eastern Mediterranean, the Lebanese mountains form a significant orographic feature in north-south-orientation making it a natural barrier and climatic transition zone between the Mediterranean Sea and the Arabian Peninsula. The study site is located at an elevation of approximately 2000m a.s.l., just below the upper tree line and experiences hot and dry summers and cool and humid winters (Köppen, 1936). In winter, precipitation at Mount al-Qurnat as-Sauda often falls as snow, which result in a yearly snow pack (Darwish, 2018). Important parts of the atmospheric oscillation, including the South Asian Monsoon, the West African Summer Monsoon, the Siberian High, the El Niño-Southern Oscillation, and the North Atlantic Oscillation have been described to influence the regional climate (Xoplaki et al., 2003).

Soils at the study site are mainly lithic leptosols with non-clastic sedimentary carbonated rocky structures which usually occur on steep slopes with angles ranging between 8° and 30° (Darwish, 2018). At the site the forest is of low density and almost exclusively dominated by *J. excelsa*, a heliophilic and orophilic tree species (Douaihy et al., 2013). Poor soil conditions, south facing slopes combined with often shade less conditions of a low-density forest were regarded as good preconditions for a high climate sensitivity at the study site (Fritts, 1976).

### 2.2 Sampling and development of ring-width and anatomical chronologies

In September 2020, 35 increment cores from 14 dominant *J. excelsa* trees were collected. Additional 18 cores from five *J. excelsa* trees were sampled in May 2021. Tree heights and diameters extended from 8 to 12 m and 40 to 80 cm, respectively, while tree ages ranged between 130 and 220 years. Sampling was conducted at breast height (1.3 m from the ground) parallel to the contour lines of the slope to avoid reaction wood, potentially present on the up- and downhill sides (Gärtner and Heinrich, 2013). A minimum of two cores were taken from each tree. Since *J. excelsa* often exhibits a strong form of lobate growth (Esper, 2000; Esper et al., 2002; Sarangzai and Ahmed, 2011), cores from each growth lobe, if possible, were taken to facilitate cross-dating. A maximum of five cores was taken from trees that presented severe lobate growth. The increment cores were prepared and then analyzed for tree-ring widths following standard dendrochronological procedures (Schweingruber, 2010; Cook and Kairiukstis, 1990; Stokes and Smiley, 1968; Fritts, 1976).

The increment cores were first surfaced carefully with a core-microtome (Gärtner and Nievergelt, 2010) and then, to increase the contrast of the ring boundaries, polished with ultrafine sandpaper up to grit size of 1200. Samples were scanned in 2400 dpi using the Epson Expression 12000XL flatbed scanner, applying a grey filter to increase the contrast of tree-ring boundaries. Finally, tree-ring width was measured using the WinDENDRO software (Regent Instruments, 2009), and the software COFECHA was used to verify dating and accuracy of measurements (Holmes, 1983). Subsequently, the raw data series were standardized and detrended using the software ARSTAN (Cook, 2002). The tree-ring width series were first power transformed to homogenize their variances. In a second step, the time series were detrended, following the approach of Touchan et al. (2007) for the same tree species comprising similar tree ages at a comparable site in the Taurus Mountains of Turkey. Each time series of tree-ring width measurements was fit with a 66% cubic smoothing spline with a 50% cutoff frequency, and each year’s ring width was divided by the year’s value of the fitted curve to give a dimensionless index with a mean of 1. This was done to remove non-climatic trends due to tree age, size, and the effects of stand dynamics (Cook et al., 1995). It was found that standardization and detrending reduced the autocorrelation substantially, and hence only first order differences were calculated for each time series of tree-ring width and wood anatomical measurements. For the site chronology building the bi-weight robust mean was preferred over the arithmetic mean because it discounts the influence of outliers (Cook, 2002).

Six cores were selected for further wood anatomical analyses. Selections criteria were samples without wood structures such as wedging rings and/or reaction wood potentially hindering the wood anatomical analysis (Carrer et al., 2017). Additional criteria were high interseries correlations and old ages to avoid ontogenetic trends, which are often found especially in the young parts of trees. The increment cores were cut into segments of approximately 5 cm lengths (Liang et al., 2013b). Prior to cutting thin sections, a non-Newtonian fluid consisting of corn starch and distilled water was applied to the surface to stabilize the cellular structures and prevent cell walls from collapsing during the mechanical procedure (Gärtner and Nievergelt, 2010; Schneider and Gärtner, 2013; Schweingruber, 2010). Rather thick transversal thin sections of 30 to 40 microns thickness were cut using a core-microtome (Gärtner and Nievergelt, 2010). Such thicker thin section were produced on purpose because they are less fragile and therefore easier to produce and handle during the following treatment steps of staining and dehydrating. Thin sections were stained with 1% safranin diluted in distilled water, rinsed and dehydrated with alcohol and mounted non-permanently between two glass slides, applying glycerol to keep the thin sections moist. The staining of the wood helps to increase the fluorescence of the wood and to enhance structural contrasts (Schweingruber, 2010). The cover glass on top ensured microscopically plane surfaces, facilitating swift microscopic imagery because the optical focus of the microscope had not to be re-adjusted when moving along the wood samples. Images were produced with a 100x optical and 3x digital magnification with an Olympus FluoView FV300 CLSM which provides high-resolution imaging through laser excitation of wood on the surfaces of the thin sections, enabling detailed observation of cellular components such as cell walls and vessels by using a projected laser beam and its reflection from the wood surface. This technique facilitated precise quantitative analysis of wood microstructures. The resulting dichromatic images (2.157 pixels/micron) were stitched with the software Photoshop Elements. Then a homogenizing filter and shade correction were performed using the Olympus *cellB* software to reduce the noise caused by reflection of the glycerol and the thickness of the section, following the protocols described in Liang et al. (2013a, b).

Cell lumen radial diameter (LD) and tangential cell wall thickness (CWT) were measured using the software ROXAS (von Arx and Carrer, 2014) supported by the software Image-Pro Plus v6.1 (“Media Cybernetics, Inc.”). Tracheidograms, namely radial profiles of wood cellular structures measured along the individual tree rings (Vaganov, 1990) were produced to extract variability of LD and CWT within and between years, and among trees. First, the R-package RAPTOR (Peters et al., 2018) was used to assign each cell a position within its respective radial file within each tree ring. At this step, the quality of measurements was assessed visually to detect any missing or misplaced values and iteratively corrected in ROXAS (von Arx and Carrer, 2014). In addition, outliers were visually checked and removed using the dplyr and lemon R packages (Edwards et al., 2020; Wickham et al., 2021). Tracheidograms for each year and tree were then computed by averaging anatomical measurements of at least 10 radial rows per tree ring, a replication exceeding the minimum recommended number of radial files (6) needed to assess intra-annual variability of xylem cellular parameters (Seo et al., 2014). Tracheidograms of single tree and single years were then divided into ten equidistant sectors, ranging from Sector I (beginning of the ring, i.e., earlywood) to X (end of the ring, i.e., latewood).

**Figure 2:**
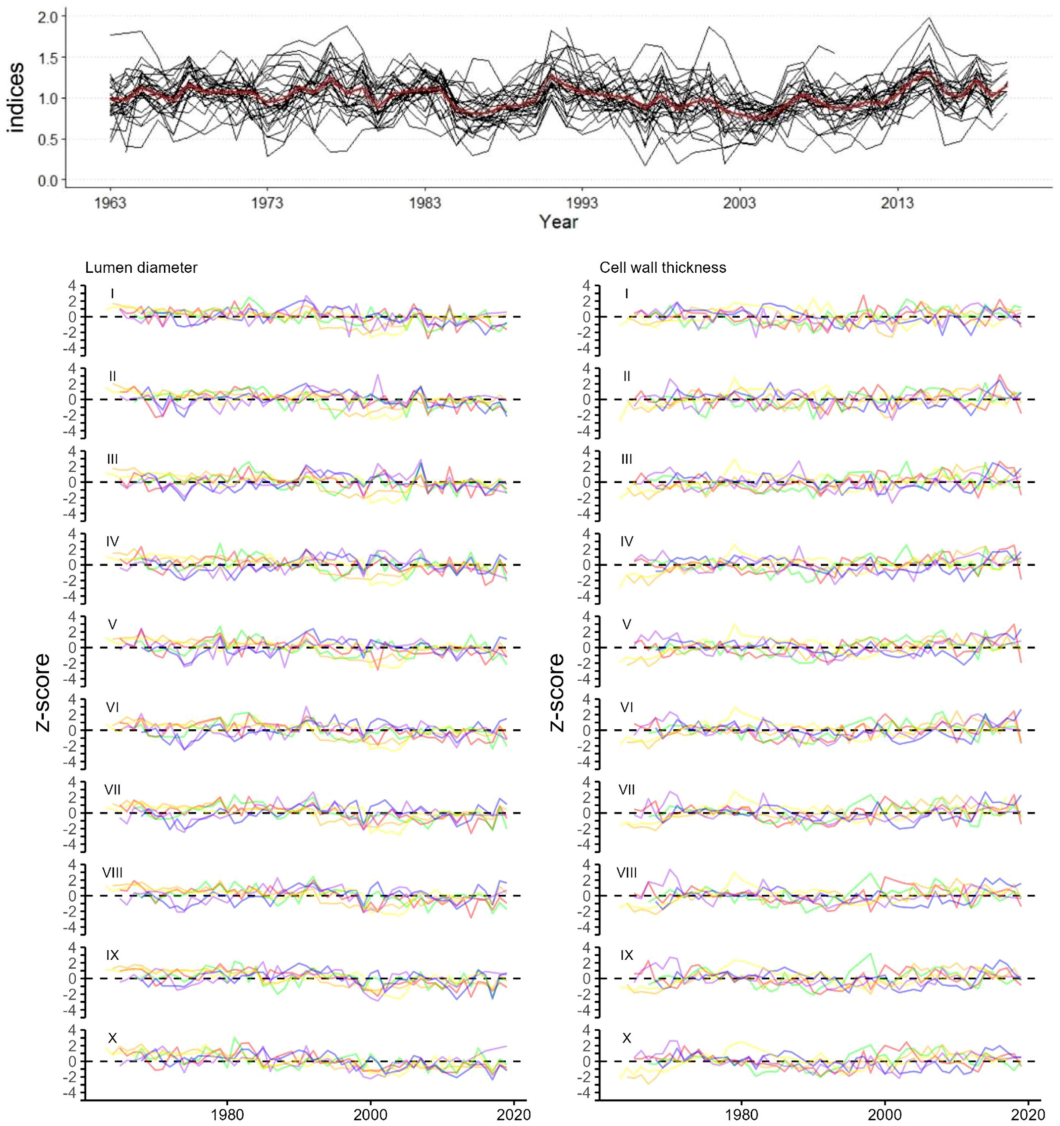
Tree-ring width indices (black) with mean (red) (top graph). z-transformed chronologies of (Bottom left) radial lumen diameter (LD sectors I to X), (Bottom right) tangential cell wall thickness (CWT sectors I to X) of 6 trees each represented by a different color. Time period for all graphs is 1963-2019.

Since the trees selected for QWA were at least 60 years older than the period of interest (years 1963 to 2019), making sure that sampling took place in the mature parts of the stems, significant long-term ontogenetic trends were not detected in the raw data (**Figure 2**, Liang et al., 2013a, b; Pritzkow et al., 2014). Further correlation analyses were conducted with unfiltered and high-pass-filtered series, calculated with first order differences, containing in the first case low- and high-frequency signals and in the second case only high-frequency signals. Measurement series were z-transformed, that is, data were standardized by converting the raw measurements into z-scores. This process involved calculating the mean and standard deviation for each individual tree-ring series and then transforming each data point by subtracting the mean and dividing by the standard deviation. This results in a dataset where each series has a mean of zero and a standard deviation of one, making them directly comparable across different trees and removing the influence of differences in overall ring size between different trees. Site chronologies for each sector were then calculated from the normalized values for each anatomical parameter for the common period of 1963 to 2019 using the arithmetic mean.

The summary statistics of the time series and measures of common signal strength were evaluated with the dplR package (Bunn, 2008) through calculated descriptive metrics commonly used in dendrochronology the mean value and the standard deviation (SD), the mean correlation between series (Rbar), the expressed population signal (EPS), signal-to-noise ratio (SNR), and the first-order autocorrelation coefficient (AR) (Bunn et al., 2021; Carrer et al., 2017). Rbar and SNR are measures of the strength of the observed common signal among the trees in a chronology. EPS indicates the extent to which the sample size is representative of a theoretical population with an infinite number of individuals (Fritts, 1976). AR is a measure of persistence of the time series and expresses the influence of previous years upon growth during the current year. Generally, Rbar, EPS and SNR are used to quantify the strength of the climate signal shared among the trees (Cook and Kairiukstis, 1990; Hughes et al., 2010; Fritts, 1976). An analysis of variance was conducted on the set of tracheidograms in all ten sectors, computed via ANOVA in R, along with box-plots for the ten sectors. Two groups were formed, Group 1 (for years 1963 to 1992) and Group 2 (for years 1992 to 2019), in order to confirm statistically the visual differences identified among the tracheidograms for the 56 years.

### 2.3 Dendroclimatic analysis

The ten sector chronologies (LD – CWT) and tree-ring-width indices (RW) were initially correlated against monthly time series of mean temperature (Tmean), maximum temperature (Tmax) and minimum temperature (Tmin), precipitation and various versions of the Standardized Precipitation-Evapotranspiration Index SPEI (ranging from the 1-month to the 24-month versions SPEI-1 to SPEI-24). The climate data, developed by Harris et al. (2020), were downloaded as gridded (0.5° resolution) monthly climate records from the KNMI Climate Explorer webpages (http://www.knmi.nl/) (Van Oldenborgh and Burgers, 2005; Trouet and Van Oldenborgh, 2013; Dataset Collection Record: Climatic Research Unit (CRU): Time-Series (TS), 2021). The parameters exhibiting the most significant correlations, that is, Tmax, precipitation and 1-month (SPEI-1) were selected for detailed analysis, presented here. The dendroclimatic analysis was performed using the Treeclim R package (Zang and Biondi, 2015) and the software Seascorr (Meko et al., 2011; Percival and Constantine, 2006) for current and previous years. Pearson correlations were computed on monthly data and monthly aggregates from 2 to 19 months. The seasonal correlation analysis was conducted with unfiltered and high-pass-filtered LD and RW series. Optimal seasonal windows with highest correlations were defined and presented. In a first try, even though the sample number was low, it was also interesting to spatially correlate our records of tree-ring widths and anatomical parameters with gridded climate data, in order to identify the geographic regions with significant correlations between climate and our tree-ring records. We used the KNMI Climate Explorer website (http://www.knmi.nl/) (Van Oldenborgh and Burgers, 2005; Trouet and Van Oldenborgh, 2013) to generate correlation fields between LD and Tmax and SPEI-1, respectively.

## 3 Results

### 3.1 Tree-ring width and cell anatomical chronologies

**Figure 3:**
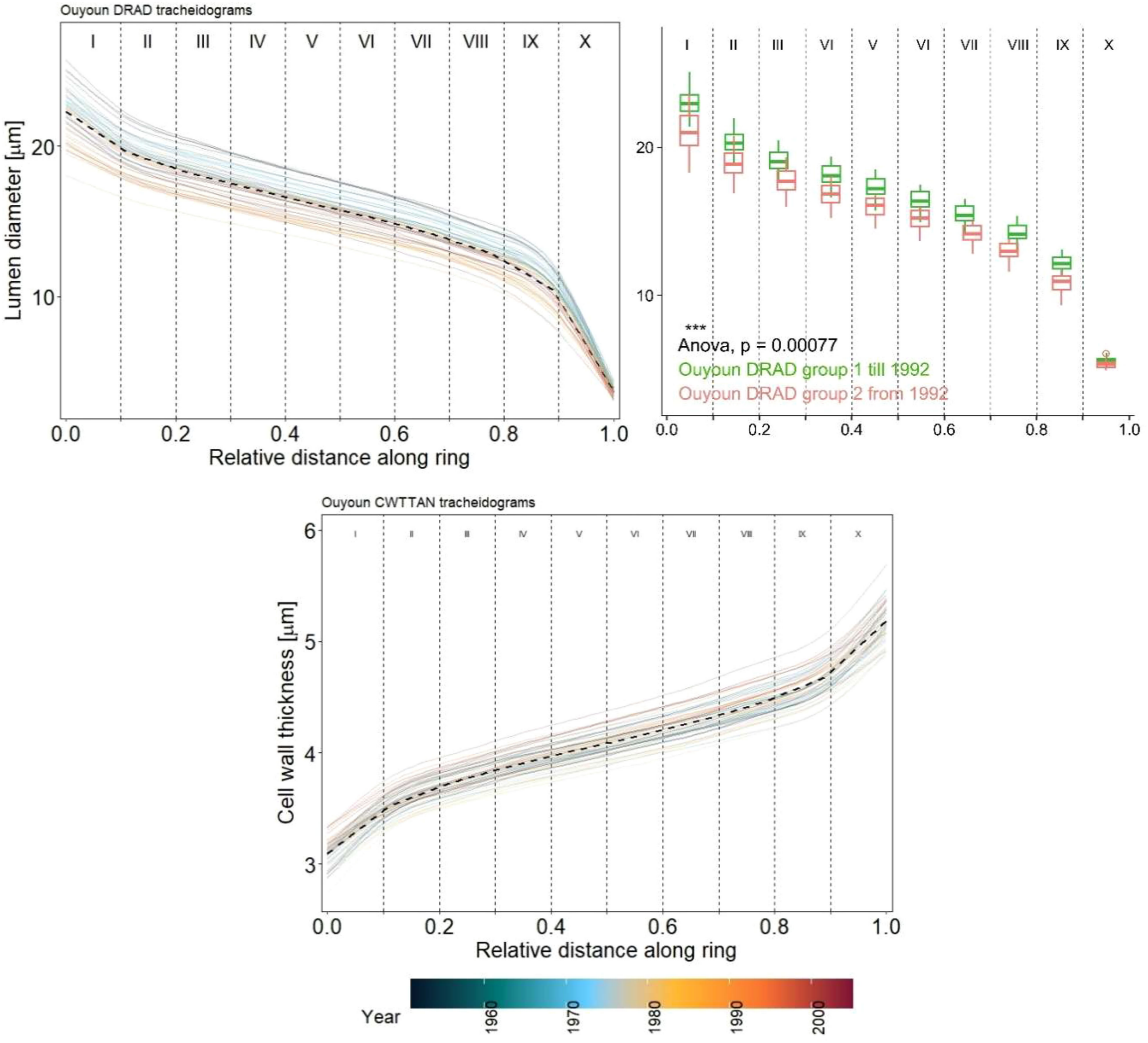
Annual colored solid lines and mean black dashed lines of standardised lumen diameter (LD) on the top, and cell wall thickness (CWT) on the bottom for the period 1963-2019. Sectors I to X represent the tree ring from the beginning to the end. The box plots divide LD into two groups (group 1 from 1963 to 1992; group 2 from 1992 to 2019), (***) indicates significant statistical difference (p ≤ 0.001) between groups as determined by ANOVA test statistics.

Tree-ring width series of *J. excelsa* displayed an Rbar of 0.429 and EPS of 0.958 for the period 1963 to 2019, revealing a strong common signal between individual trees (**Table 1**; **Figure 2**). Generally, chronology statistics were notably higher for LD than for CWT. Within the ten sector chronologies of LD, the statistics for Rbar, EPS, and SNR were highest in the last sectors IX and X, that is, towards the end of the ring. All statistics were well in the range of acceptable benchmarks, indicating strong common signals in all sectors, however, minor disharmonies were exhibited in sectors IV, V and VI, also indicated by lower EPS and Rbar values. CWT exhibited a weak common signal between the six trees across all sectors (**Table 1**; **Figure 2**). The average values for LD decreased from 22.4 ± 1.63 µm for sector I to 3.7 ± 0.31 µm for sector X while the CWT values increased from 3.1 ± 0.13 µm for sector I to 5.2 ± 0.18 µm for sector X (**Table 1**; **Figure 3**). Since the descriptive statistics for CWT were very low, in the following climate-growth correlation analyses it was included only as a reference.

**Table 1:** Descriptive statistics for normalized cell radial lumen diameter (LD), tangential cell wall thickness (CWT) sector chronologies and ring-width (RW); Mean & SD (Mean ± standard deviation, in µm), AR (autocorrelation of the series with 1 year lag), Rbar (mean correlation among individual trees series), EPS (expressed population signal), and SNR (signal-to-noise ratio).

RW	Raw RW chronology statistics					Standardised RW chronology statistics				
	SD	AR	mean Rbar	EPS	Std error	SD	AR	mean Rbar	EPS	Std error
0.251	0.922	0.415	0.955	0.011	0.248	0.234	0.429	0.958	0.012
Sector	Lumen diameter LD					Cell wall thickness CWT				
	Mean & SD	AR	Rbar	EPS	SNR	Mean & SD	AR	Rbar	EPS	SNR
I	22.4 ± 1.63	0.55	0.283	0.703	2.678	3.1 ± 0.13	0.60	0.022	0.121	0.204
II	19.7 ± 1.23	0.50	0.293	0.714	2.642	3.5 ± 0.10	0.61	0.095	0.39	0.356
III	18.3 ± 1.13	0.26	0.301	0.721	2.718	3.7 ± 0.10	0.63	0.039	0.201	0.225
IV	17.3 ± 1.08	0.31	0.190	0.583	1.420	3.9 ± 0.11	0.66	0.035	0.182	0.191
V	16.3 ± 1.04	0.43	0.241	0.656	1.567	4.0 ± 0.11	0.53	0.007	0.048	0.136
VI	15.3 ± 1 .00	0.47	0.249	0.665	1.734	4.1 ± 0.11	0.57	0.052	0.250	0.145
VII	14.3 ± 0.96	0.48	0.305	0.725	2.223	4.3 ± 0.12	0.49	0.06	0.280	0.322
VIII	12.9 ± 0.94	0.53	0.288	0.709	2.168	4.4 ± 0.13	0.47	0.024	0.131	0.239
IX	10.5 ± 0.95	0.59	0.379	0.786	3.308	4.7 ± 0.14	0.26	0.077	0.337	0.725
X	3.7 ± 0.31	0.67	0.389	0.793	3.138	5.2 ± 0.18	0.36	0.062	0.289	0.458
Mean	15.07± 1.03	0.48	0.2918	0.7055	2.3596	4.09 ± 0.123	0.52	0.0473	0.223	0.3001

The series span from 1963 to 2019. Sector I is the first percentile of the series and represents the start of the earlywood part of the ring, whereas sector X is the last percentile of the data or end of the latewood part of the ring.

CWT values were closely distributed around the mean and increased evenly from sector II to IX, while a sharper increase between sectors I-II and IX-X was noticeable (**Figure 3**). Similarly, LD values were closely distributed around the mean. However, LD decreased uniformly from sector II to VIII with a sharp decrease between sectors I and II and sectors VIII and IX. The sharp decrease of LD and increase of CWT in the last sectors, i.e., sectors IX to X, reflects the latewood part of the tree rings in juniper, with very small cell lumen and thick cell walls. The distribution of the annual values around the mean highlights a striking change of LD in the early 1990s and CWT in the late 1990s (**Figure 3**). LD values have been below average for nearly all years since the early 1990’s whereas prior to that period, they consistently remained above average. This difference showed a high statistical significance (p=0.00077) between Group 1 (for years 1963 to 1992) and Group 2 (for years 1992 to 2019) when tested with ANOVA (**Figure 3**). The CWT distribution showed a similar pattern with a 7-year delay (*p* = 0.014) between the two groups.

### 3.2 Correlation with monthly climate

**Figure 4:**
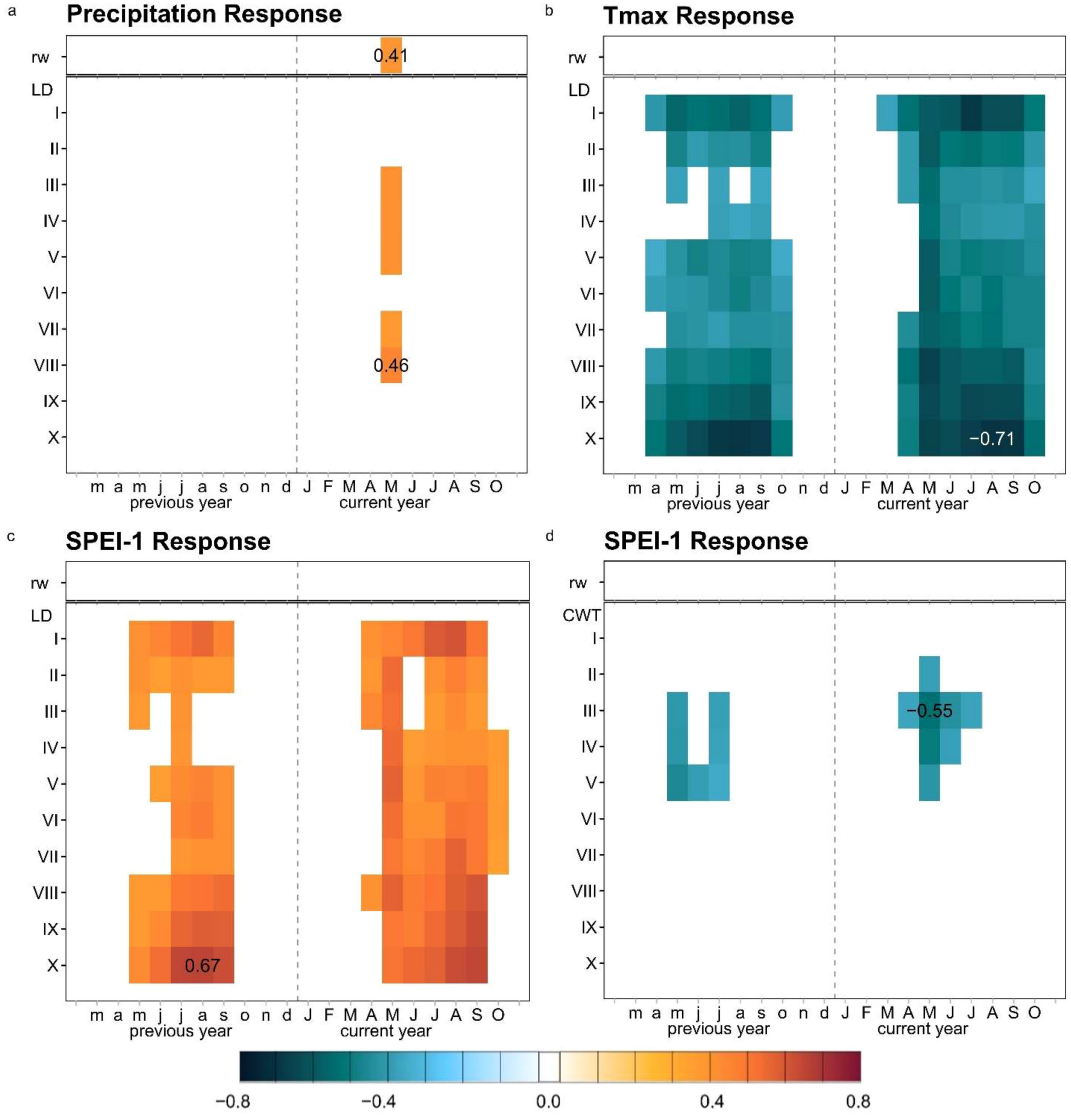
Monthly correlations of tree-ring-width indices (rw), radial lumen diameter sectors (LD I-X), and tangentiel cell-wall thickness sectors (CWT I-X), for the period 1963-2019; **(a)** with monthly averaged precipitation; **(b)** with monthly averaged maximum temperature (Tmax); **(c, d)** with SPEI-1. Each colored tile represents significant correlations at p ≤ 0.05, with the highest correlation value plotted on the corresponding tile of each heatmap.

RW exhibited a significant positive correlation with current May precipitation (r = 0.41) but correlations with Tmax and SPEI-1 could not be identified (**Figures 4 a-d**). In contrast, wood anatomy chronologies, in particular LD, correlated well with Tmax and SPEI-1 (**Figures 4a-d**). Almost all sectors for LD showed negative correlations with Tmax and positive correlations with SPEI-1 during the growing season (May to Sep), always following similar patterns. Significant correlations, particularly for the period July-to-September of current and previous year of growth, were concentrated in the latewood and earlywood part of the tree-ring patterns (**Figures 4b-d**). The highest correlation values between LD and climate variables were found in Sector I, II, VIII, IX and X, the maximum value was identified for sector X (r = -0.71) (**Figure 4b**). LD sectors VII and VIII displayed positive correlations with current-May precipitation, with a maximum in sector VIII (r = 0.46).

### 3.3 Seasonal correlations

**Figure 5:**
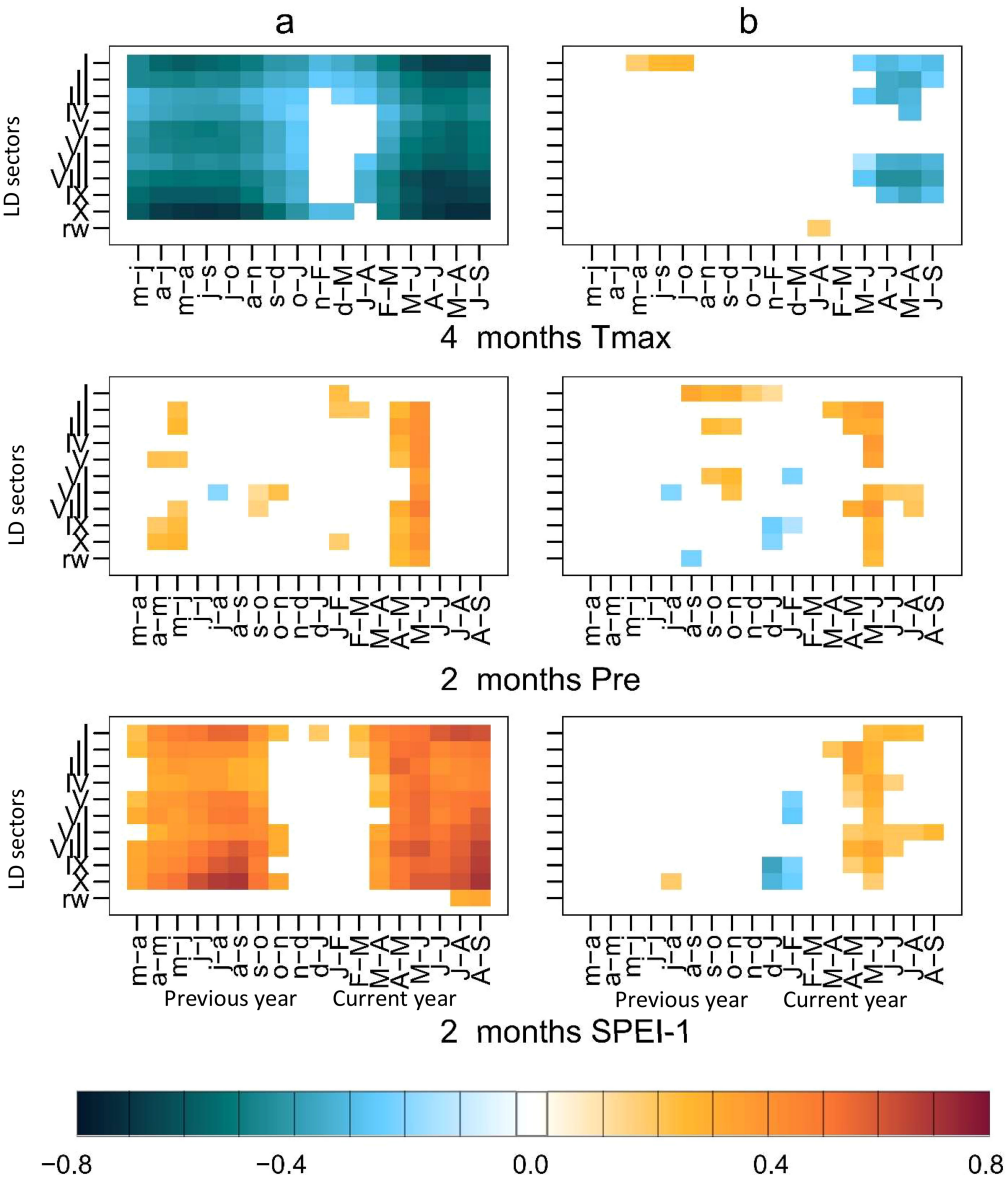
Seasonal correlations [seascorr (Meko et al., 2011)] for the period 1963-2019. Each coloured tile represents a significant correlation at p ≤ 0.05. On the y-axis, the lumen diameter chronologies LD sectors I to X and tree-ring-width indices (RW). On the x-axis, the climate season for each parameter is displayed, with the small letters indicating 1 year lag, and the capital letters representing the current year. Unfiltered data shown in **(a)** and, high-pass-filtered data shown in **(b)** for the same climate seasons. Detailed information on the optimal correlation seasons can be found in **Table 2**.

**Figure 6:**
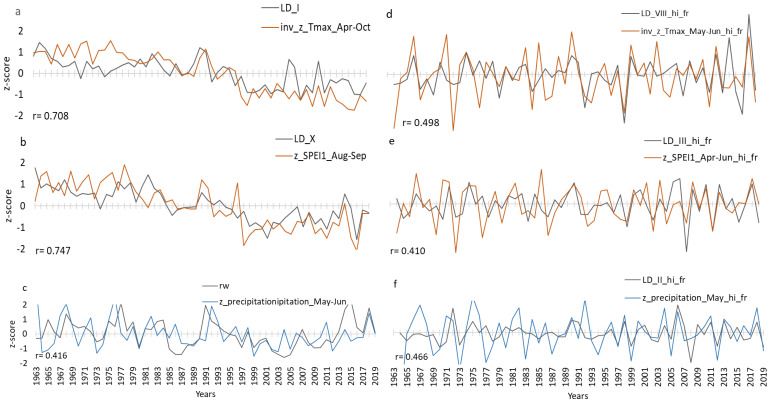
Comparison plots for the period 1963–2019 showing optimal seasonal correlation **(a–c)** between normalised (z_scored) LD sector-I in black and inverse Apr-Oct Tmax in orange **(a)**; LD sector-X in black and Aug-Sep SPEI-1 in orange **(b)** and RW in black and May-Jun Precipitation in blue **(c)**. Panels d-f represent the high-pass filtered series: LD sector-VIII in black and inverse May-Jun Tmax in orange **(d)**, LD sector-III in black and Apr-Jun SPEI-1 **(e)**, LD sector-II in black and May Precipitation in blue **(f)**. On the x axis are years and on the y axis normalized unitless values of the respective climate and tree ring parameters. “r” is the Pearson correlation coefficient with the significance level being r=0.25 at p=0.05.

To further study the control of temperature and precipitation on the lumen diameter LD, seasonal correlations between LD sectors I to X and RW were calculated for the period 1963 to 2019 comprising seasons ranging between 2 and 19 months and starting from March of previous year to October of current year (**Figure 5**). The results for the z-transformed data (**Figure 5a**) and high-pass-filtered data (**Figure 5b**) show differences, especially for Tmax and SPEI-1, suggesting stronger correlations in **Figure 5a**. Correlations between LD and precipitation were generally lower and limited to the seasonal climate from April to June of the current year with only little previous-year effects. In contrast, correlations with Tmax and SPEI-1 showed clear previous-year effects (**Figure 5a**). Since the z-transformed data exhibited the stronger correlations, in a next step, strongest seasonal correlations were selected for further graphical comparisons (**Figure 6**), separated for z-transformed data (**Figures 6a–c**) and high-pass-filtered data (**Figures 6d–f**). In particular, the z-transformed LD data for Sector I and Sector X exhibited strong correlations with April-to-October Tmax and August-to-September SPEI-1 (**Figures 6a, b**), respectively, obviously related to common trends found in the LD time series as well as in Tmax and SPEI-1 data.

**Table 2:** Optimal seasonal correlations between the 10 sectors of lumen diameter chronologies (LD I to LD X), tree-ring-width indices (RW) and climate parameters Tmax, Precipitation (Prec.) and SPEI-1.

**a**												
Chronology		LD I	LD II	LD III	LD IV	LD V	LD VI	LD VII	LD VIII	LD IX	LD X	RW
Tmax	season	Apr-Oct	Apr-Jul	Apr-Jun	May-Oct	May-Oct	May-Jun	May-Jun	Apr-Sep	Apr-Oct	Jul-Oct	Jul-Aug
	corr	**-0.708**	-0.612	-0.54	-0.514	-0.581	-0.603	-0.615	-0.692	**-0.699**	**-0.759**	-0.27
Prec.	season	May*-Mar	May-Jun	May-Jun	May-Jun	May-Jun	May	May-Jun	May-Jun	May-Jun	May-Jun	May-Jun
	corr	0.401	0.346	0.373	0.366	0.37	0.334	**0.405**	**0.43**	0.348	0.349	**0.416**
SPEI-1	season	Jul-Sep	May-Oct	Apr-Sep	May-Oct	May-Sep	May-Oct	May-Oct	Aug-Sep	Aug-Sep	Aug-Sep	Aug-Sep
	corr	0.667	0.566	0.542	0.549	0.598	0.598	0.626	**0.684**	**0.707**	**0.747**	0.362

**b**												
Chronology		LD I	LD II	LD III	LD IV	LD V	LD VI	LD VII	LD VIII	LD IX	LD X	RW
Tmax	season	Mar*-Oct*	May-Jun	Apr-Jun	May-Jun	May	May-Jun	Jun	May-Jun	May-Jun	Jan	~
	corr	0.364	**-0.427**	-0.409	**-0.432**	-0.286	-0.342	-0.415	**-0.498**	-0.404	0.274	~
Prec.	season	Sep*-Mar	May	May-Jun	May-Jun	May-Jun	Sep*-Nov*	May-Aug	May-Sep	May-Sep	Dec*	May-Sep
	corr	0.39	**0.466**	0.394	**0.464**	0.364	-0.296	0.412	**0.454**	0.349	-0.315	0.328
SPEI-1	season	Mar-Sep	Apr-Jun	Apr-Jun	May-Jun	May	~	Apr-Aug	Apr-Sep	Dec*-Jan	Dec*-Jan	~
	corr	0.354	**0.394**	**0.41**	0.338	0.319	~	0.313	**0.358**	-0.385	-0.338	~

Significant Pearson correlations (p=0.05). Negative correlations are depicted in blue while positive in orange. The three highest correlations are in bold. “Month*” represents previous year and “Month” current year. (a) for unfiltered and (b) for high-pass filtered climate parameters and LD chronologies.

### 3.4 Spatial correlations

**Figure 7:**
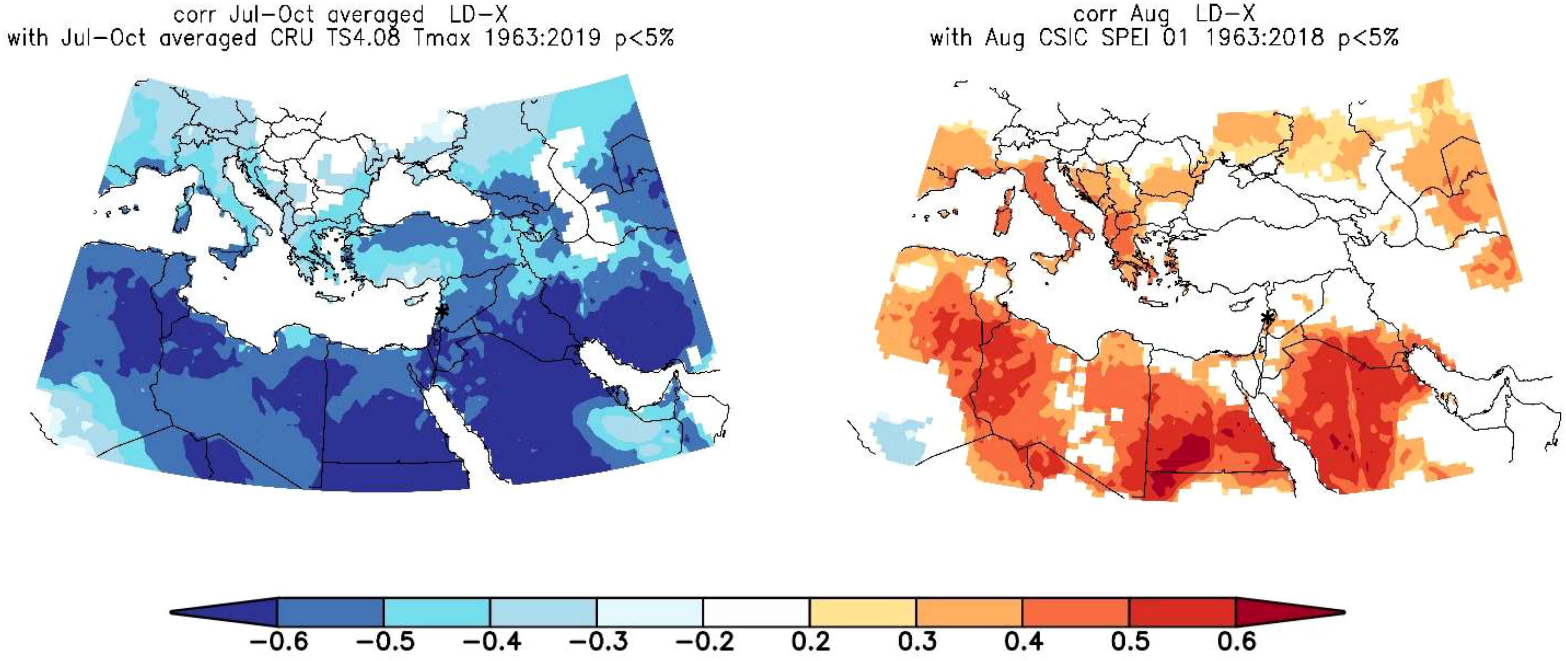
Spatial field correlation showing the relationship between Sector X lumen diameter and July-October Tmax (left) August SPEI-1 (right). Shaded areas are significant correlations at p < 0.05, * is the site location.

LD chronologies showed strong correlations with SPEI-1 and Tmax, hence we decided to conduct spatial correlations focusing on these two climate parameters. Spatial correlations were calculated for an area covering the MENA region (10°W to 50°E and 10°N to 40°N) (**Figure 7**). The spatial correlation patterns of LD with SPEI-1 and Tmax are spatially relatively consistent in large parts of the MENA region (**Figure 7**). While correlations between lumen diameter and Jul-Oct Tmax were negative, with large areas indicating field correlations of r = -0.6 and higher in the entire MENA region, correlation patterns between lumen diameter and Aug SPEI-1 were positive, with r-values ranging mainly between 0.4 and 0.6 especially in regions of North Africa. Overall, correlations were particularly strong in southern MENA, suggesting that climate patterns from the south exert a greater influence on tree growth during the growing season than the westerly and northerly climate.

## 4 Discussion

The summary statistics describing the common growth patterns of *J. excelsa* tree-ring width series in Lebanon for the period 1963 to 2019 indicated solid correlations (e.g., EPS = 0.95 and Rbar = 0.429). Similar values were reported in Köse et al. (2011) for southern Turkey, and were used to justify the usefulness of the site for further dendroclimatological investigations. In our study lumen diameter series also showed some common variability reflected by Rbars ranging from 0.3 to 0.4 in most sectors of the ring. Similar Rbars of 0.25 to 0.4 for LD were presented in comparable studies (Fonti et al., 2014; von Arx et al., 2016; Carrer et al., 2017). Even though EPS values for our LD series were lower than 0.85 (~0.79 in most sectors), 0.85 being regarded as a critical lower limit in dendrochronology (Wigley et al., 1984), reliable climate signals could be identified. Similar experiences were reported by Carrer et al. (2017) which led to the argument that the EPS threshold, originally developed for tree-ring width series, may not be appropriate or necessary for wood anatomical time series. It was then discussed if the lower values were due to a higher biological variability in wood anatomical time series than is usually found in tree- ring width.

While LD data exhibited promising interseries and climate-growth correlations, we found that sector chronologies of cell wall thickness (CWT) had little common variability, expressed by low values of Rbar and EPS. This lack of common signal in cell wall properties had been found previously at other drought-sensitive locations and ascribed to the high variability of carbon assimilation and allocation dynamics among individuals (Balanzategui et al., 2021; Peltier and Ogle, 2020; Ziaco et al., 2016). Additionally, we noted that cell lumen of *J. excelsa* exhibited significantly greater variation than did cell wall thickness, in particular in radial direction, which indicated more sensitivity and that LD is the more promising parameter in quantitative wood anatomy for future dendroclimatic research. By increasing the number of measured tracheids, the chronology statistics and the proportion of common signals could be improved (Bryukhanova and Fonti, 2013).

Our findings showed that intra-annual patterns of tree anatomical parameters, such as radial diameter of cell lumen and tangential thickness of cell walls reflect adaptations by *J. excelsa* to maintain hydraulic integrity during the growing season, in particular to withstand rising temperatures and increasing drought conditions typical for the subtropics, as has been suggested by Bryukhanova and Fonti (2013). The observed patterns of dendroclimatic correlations suggest that the response of *J. excelsa* to increasingly dry conditions is primarily modulated by adjustments in the lumen diameter, as has been reported for other conifers growing in water limited environments (Castagneri et al., 2018; Balanzategui et al., 2021).

One major finding is that inter-annual variations of LD presented by sets of tracheidograms show a distinct change of trends over the study period around the year 1992, with above-average values from 1963 to 1992 and below-average values from 1992 to 2019 (**Figure 3**). This division between early and late years corresponds with significant increases in maximum temperature and drought in the early 1990s, likely marking the onset and regional expression of global warming, which was corroborated by the same trends in the regional climate data (Tmax and SPEI-1) and had already been identified by Dogan et al. (2015) (**Figures 6a, b**). LD follows the decadal temperature and drought trends suggesting that low-frequency climate signals will be reflected in long-term reconstructions based on LD measurements. Since 1992, LD has been decreasing, reflecting an adaptation to increasing heat and drought in the MENA region. This adaptation still seems to work as *the* sp*ecies* has been able so far to decrease LD each year in response to the increasingly adverse climatic conditions, however, there are always limits to the wood anatomical plasticity of tree species (Eilmann et al., 2009; Matisons et al., 2019; Fontes et al., 2022). The results also suggest that future studies, aiming to use wood anatomical series of *J. excelsa* for long-term climate reconstructions, need to be careful when detrending data series before dendroclimatological analysis because low-frequency climate signals might also be partly lost and with it the ability to reconstruct low-frequency variations.

The time series of *J. excelsa* from Lebanon seem to support this argument, but more sites and samples are needed to confirm the initial findings.

Sectoring the tree rings allowed for a more accurate capturing of seasonal correlations, providing insights into seasonal growth of *J. excelsa* and how it was related to seasonal climate patterns. In particular, May-to-September Tmax and SPEI-1 of both the current and previous year emerged as the most limiting factors for LD of the early and late parts of the ring (i.e., sectors I, II and VIII, IX, X). These climate-tree-growth correlations can likely be ascribed to the abrupt change in hydroclimate dynamics happening every year which are caused by the “Sirocco” winds blowing from the Sahara in early spring, while in winter and spring the study region is usually dominated by the effect of the cold northerly air masses called “Mistral” and “Bora” (Sivall, 1957).

Certainly, the incoming hot southern air masses drive the temperatures up, melt snow-pack and initiate the growing season (Sivall, 1957; Darwish, 2018). This is confirmed by the response to May temperature and SPEI-1 of the current year across all lumen diameter sectors of the whole ring (**Figures 4b, c**). The same pattern holds true for precipitation, with significant May and May-to-June precipitation correlations with LD sectors II to X (**Figures 4**, **5**). This study corroborates previous studies in the region, which showed that precipitation in May exerts the most significant impact on tree-ring width of *J. excelsa* (Touchan et al., 1999). Similarly, correlations between coniferous wood anatomical parameters and temperature and precipitation were reported by (Bryukhanova and Fonti, 2013). However, neither Touchan et al. (1999) nor the current study identified significant correlations between tree-ring width and temperature. In this study, only LD correlated with Tmax, which suggests that *J. excelsa* is sensitive to evapotranspiration conditions, but responds to temperature variability by adjusting its lumen diameter rather than cell wall thickness (**Figures 4**, **5**). Similar results were previously reported for Douglas fir growing under climate conditions similar to those of juniper in the current study (Peltier and Ogle, 2020; Balanzategui et al., 2021).

In a final step, we also examined the correlation of LD with gridded July-to-October Tmax and August-SPEI-1 data, in order to identify the geographic spread of the strongest seasonal correlations. Generally, regional climate is influenced more or less by various climate forcing and teleconnection patterns which are all part of the global atmospheric circulation, including e.g. Asian and African Monsoon, Siberian High, Arctic Oscillation and North Atlantic Oscillation (Xoplaki et al., 2003). Spatial field correlations indicate that cell lumen data correlate more with the Tmax and SPEI-1 fields in the South and Southeast (**Figure 7**), suggesting that climate patterns that limits tree growth at our study site are more linked to southern components of the climate system (i.e., Monsoon, ENSO) (Xoplaki et al., 2003). According to (Alpert et al., 2004) several large synoptic situations, e.g. the Cyprus low and Red Sea trough, have been identified important for climatic variations in the eastern Mediterranean. Climatic trends, which are also suggested by our tree ring record, might be related to changes in such synoptic situations (Alpert et al., 2004). Furthermore, the regional climate is influenced by the Mediterranean Sea itself (Trigo et al., 1999; Mariotti et al., 2002). Since the Mediterranean Sea is experiencing significant warming trends (Kubin et al., 2023), the climate dynamics at our study site are likely altered by this situation. Atmospheric blocking and subtropical air intrusions from the Sahara have been identified as critical drivers of extreme temperature events, such as heatwaves in the Mediterranean (Sousa et al., 2020; Cos et al., 2024). Since our new proxy record suggests to have wide regional correlations in MENA, further research should focus on using lumen diameter. However, confirmation through sampling and combining more sites in the region is needed.

## 5 Conclusions

We demonstrated for the first time that quantitative wood anatomy can be successfully applied to *J. excelsa* wood to extract long-term growth trends and valuable climatic information. The results presented here indicate that the interannual variability of tracheid lumen diameter is the most signal-rich parameter, and that the climatic signals in wood anatomical structures, in particular in lumen size, differ from that found in tree-ring widths, and are statistically stronger. Therefore, lumen diameter should be regarded as a valuable new proxy to retrieve additional and more significant paleoclimate data, offering greater precision in reconstructing seasonality of past climate while preserving the footprint of low-frequency climatic fluctuations in the MENA region.

Our study also revealed that lumen diameter of *J. excelsa* at this site in Lebanon has been declining since the early 1990’s suggesting a bleak future for these forests in the MENA region under threat of global warming impacts. Further research is needed, including studies on daily correlation patterns and sampling at additional sites covering different elevations, slope angles and aspects in Lebanon Mountains and the region, to confirm our findings and to shed more light on the declining trends observed in LD since the early 1990s.

## Data Availability

The raw data supporting the conclusions of this article will be made available by the authors, without undue reservation.
